# Supplementary material for: High-intensity eccentric hip abductor strength training improves dynamic knee valgus in a task-dependent manner in asymptomatic young women: A pilot study
Source: PLoS One. 2026 May 5;21(5):e0330140. doi: 10.1371/journal.pone.0330140 (PMC13143104; doi:10.1371/journal.pone.0330140)
Supplement: Attachment — Submitted filename: Response to Reviewers2026_01_06.docx [file pone.0330140.s003.docx]

**RESPONSES TO EDITOR AND REVIEWERS**

Manuscript ID: PONE-D-25-39091

Dear Editor,

Thank you for handling the reviewing process and for the comments. Please find below our point-by-point responses:

**Response to the Editor’s comments:**

**Comment 1**

Please ensure that your manuscript meets PLOS ONE's style requirements, including those for file naming.

**Response to comment 1**

The manuscript has been formatted according to the linked formatting criteria.

**Comment 2**

Please state what role the funders took in the study.  If the funders had no role, please state: "The funders had no role in study design, data collection and analysis, decision to publish, or preparation of the manuscript." If this statement is not correct you must amend it as needed.

Please include this amended Role of Funder statement in your cover letter; we will change the online submission form on your behalf.

**Response to comment 2**

Funders had no role in study design, data collection and analysis, decision to publish, or preparation of the manuscript. We also make this statement in the cover letter.

**Comment 3**

We note that your Data Availability Statement is currently as follows: [All relevant data are within the manuscript and its Supporting Information files.]

Please confirm at this time whether or not your submission contains all raw data required to replicate the results of your study. Authors must share the “minimal data set” for their submission. PLOS defines the minimal data set to consist of the data required to replicate all study findings reported in the article, as well as related metadata and methods (<https://journals.plos.org/plosone/s/data-availability#loc-minimal-data-set-definition>).

For example, authors should submit the following data:

- The values behind the means, standard deviations and other measures reported;

- The values used to build graphs;

- The points extracted from images for analysis.

Authors do not need to submit their entire data set if only a portion of the data was used in the reported study.

If your submission does not contain these data, please either upload them as Supporting Information files or deposit them to a stable, public repository and provide us with the relevant URLs, DOIs, or accession numbers. For a list of recommended repositories, please see <https://journals.plos.org/plosone/s/recommended-repositories>.

If there are ethical or legal restrictions on sharing a de-identified data set, please explain them in detail (e.g., data contain potentially sensitive information, data are owned by a third-party organization, etc.) and who has imposed them (e.g., an ethics committee). Please also provide contact information for a data access committee, ethics committee, or other institutional body to which data requests may be sent. If data are owned by a third party, please indicate how others may request data access.

**Response to comment 3**

The minimal dataset required is available on the Zenodo platform under the following DOI:https://doi.org/10.5281/zenodo.1808373 .

This access location is indicated in the Data Availability Statement section of the manuscript, between lines 395–398.

**Comment 4**

PLOS requires an ORCID iD for the corresponding author in Editorial Manager on papers submitted after December 6th, 2016. Please ensure that you have an ORCID iD and that it is validated in Editorial Manager. To do this, go to ‘Update my Information’ (in the upper left-hand corner of the main menu), and click on the Fetch/Validate link next to the ORCID field. This will take you to the ORCID site and allow you to create a new iD or authenticate a pre-existing iD in Editorial Manager.

**Response to comment 4**

Thank you for calling attention. We have created the ORCID ID for the corresponding author.

ORCID iD: 0009-0007-2246-0883

ORCID record: <https://orcid.org/0009-0007-2246-0883>

**Comment 5**

If the reviewer comments include a recommendation to cite specific previously published works, please review and evaluate these publications to determine whether they are relevant and should be cited. There is no requirement to cite these works unless the editor has indicated otherwise.

**Response to comment 5:**

The reviewers had no recommendations to cite specific works.

**Response to reviewers’ comments**

**Responses to ScreenIT report (Reviewer 1) comment**

**Comment 1:**

Flow charts and attrition

We did not find a study flow chart of excluded observations. We strongly recommend using flow charts because they provide an overview of the study design and more information about attrition. If you included a study flow chart in your supplemental files, we apologize for missing this. Our tool is not able to screen separate supplemental files.

Sentence about attrition: not detected. Please provide information about the drop-out of subjects, or loss of animals or samples. This could be done using a study flow chart, or described in the text.

**Response to comment 1**

The study flow chart was already uploaded among the submitted files as Figure 1, presenting the participant inclusion and exclusion process.

There was no attrition during the study, and we state this in the Materials and Methods section on line 130.

**Comment 2**

Randomization

Not detected. If you performed an experimental study, please specify whether participants, animals or samples were randomly assigned to treatments or groups, and specify the randomization procedure.

**Response to comment 2**

We applied the Z-score method, which is a directed group allocation procedure. The description of this method can be found in lines 142-144 of the manuscript. We made small modifications in the manuscript text. The essence of our method is to ensure that the baseline values of the measured variables did not differ between the two training groups. Statistical results showing the comparison of the baseline values are presented in lines 144-147).

**Comment 3**

Blinding

Not detected. Please specify whether blinding was used at various phases of the experiment (e.g., blinding of patients, caregivers, outcome assessments, data analysis).

**Response to comment 3**

We have supplemented the Experimental Procedure session with the following sentences: „Participants were unaware about the identity of their matched partner in the other experimental group. Also, participants were informed about their individual improvements only after the completion of the study

**Comment 4**

Power Analysis

Not detected. Please state how you determined the sample size for the study. If you performed a power or sample size calculation, please provide details.

**Response to comment 4**

Because this was a pilot study, according to the journal’s policy, formal power calculation was not required.

**Comment 5**

Open code

If you wrote code to analyze your data, please consider sharing the code in a public repository. This makes it easier for others to reproduce your analyses, and may also aid others seeking to analyze similar datasets.

**Response to comment 5**

We did not develop any specific computer program and did not use any computer codes to reduce and analyse data. We used cursor-driven methods to manually analyse the data. For statistical followed only standards statistical procedures.

**Comment 6**

Open data

If permitted, sharing data on a public repository with a persistent identifier (a DOI or accession number) can improve reproducibility and make it easier for other scientists to expand on your work. Papers with open data are cited more often than papers without open data. Some institutions have an expert who can provide advice on data sharing.

**Response to comment 6**

As noted above under ‘Response to Editor’s comment 3’, we have uploaded an excel sheet with the raw data in the Supporting Information Files

**Responses to reviewer 2**

**Comment 1**

Hello to all authors. Was this pilot study conducted to assess participants’ acceptance of the protocol, to evaluate the validity of measurement tools, or to estimate the dropout rate for a future main study?

**Response to comment 1**

The study aimed to compare the effects of a four-week-long dynamometric concentric vs. eccentric hip abduction exercise training on eccentric hip abduction peak torque, magnitude of dynamic knee valgus, and single leg counter-movement jump mechanical impulse in healthy young females diagnosed with dynamic knee valgus. Therefore, this study was not designed specifically to assess participants’ acceptance of the protocol, to evaluate the validity of measurement tools, or to estimate the dropout rate for a future main study. We note that after enrolment, no participant has dropped out, implying 100% acceptance of the protocols and 0% dropout. Results from this pilot study offer valuable preliminary evidence supporting the extension of the protocol into the clinical field.
