# Supplementary material for: High-intensity eccentric hip abductor strength training improves dynamic knee valgus in a task-dependent manner in asymptomatic young women: A pilot study
Source: PLoS One. 2026 May 5;21(5):e0330140. doi: 10.1371/journal.pone.0330140 (PMC13143104; doi:10.1371/journal.pone.0330140)
Supplement: Attachment — Submitted filename: Response to Reviewers.docx [file pone.0330140.s004.docx]

**RESPONSES TO EDITOR AND REVIEWERS**

Manuscript ID: PONE-D-25-39091

Dear Editor,

Thank you for handling the reviewing process and for the comments. Please find below our point-by-point responses:

**Response to reviewers’ comments**

**Responses to Reviewer #3 Comments**

General Overview:
This pilot study investigates a relevant and timely research question: whether the mode of hip abductor strength training (eccentric vs. concentric) differentially affects dynamic knee valgus (DKV) and jump performance in asymptomatic young women. The study is well-motivated, highlighting the often-overlooked eccentric control function of the hip abductors. The use of an isokinetic dynamometer for precise, maximal-effort training and testing is a significant strength. The findings that eccentric training was superior in reducing DKV during a single-leg countermovement jump (SLCMJ) but not during a drop landing are novel and interesting. However, several methodological ambiguities, statistical reporting issues, and overinterpretations in the discussion need to be addressed before the manuscript is suitable for publication.

**Response to general overview**

Dear Reviewer,

We appreciate your general positive feedback on the research study desing and importance, and we are grateful for your critical comments.

Major Concerns:

**Comment 1  (Participant Selection and DKV Quantification)**

The inclusion criterion of "presence of DKV subjectively evaluated by a physiotherapist using the single leg squat test" is a major methodological weakness. Subjective visual assessment is known to have limited reliability. To strengthen the study's validity, the authors should provide data on the inter- or intra-rater reliability of this assessment. Furthermore, since baseline DKV values were subsequently measured with 3D motion capture, why were these objective data not used as the primary inclusion criterion? A minimum threshold of DKV (e.g., >10°) would have created a more homogeneous and relevant sample.
**Response to comment 1**

We understand your point. The subjective evaluation of DKV by a physiotherapist was applied as a pre-check for convenience: giving either “exclude” or “include”, therefore inter-rater reliability would have not made sense here. In fact, one IMU measurement session (involving setup, calibration, CMJs and landings) lasted approximately 45 min, and for simple ethical reasons we did not intend to expose volunteers to such measurement procedure, and excluding them afterwards. After the subjective pre-check, we performed the actual DKV measurements with the IMU sensors and found that all participants reached a threshold of 8° at least in one of the two test, we did no further exclusions. Up to now there is no general consensus on the exact DKV threshold for ACL injury, one recent study(Li & Qian, 2025) suggests 7-9° DKV angle as threshold. So we can conclude, that the physiotherapist’s subjective evaluation seemed a valid pre-check. We clarified this process in the manuscript in section “participants” as follows: “When the actual quantitative DKV measurements were preformed, all participants demonstrated greater than 8° of DKV in at least one of the two DKV tests. Considering recent literature suggesting 7-10° as thresholds for ACL injuries, we made no further exclusions.”

**Comment 2 (Ecological Validity of the Training Intervention)**

While the use of an isokinetic dynamometer allows for excellent experimental control, its ecological validity is a concern. Training the hip abductors in a side-lying, non-weight-bearing position is quite different from the functional, weight-bearing context in which DKV occurs (e.g., jumping and landing). The authors should discuss this as a key limitation. The impressive strength gains (20-42%) may be specific to this testing/training apparatus and may not fully translate to the coordinated, multi-joint control required during the SLCMJ and landing tasks.
**Response to comment 2**

Expecting transfer effect in a functional task while exercising with a non-functional task is not unique in physical activity, especially in resistance training. The concept is that hip abductors can be exposed to high intensity stimuli only in non-weight-bearing positions (Boren et al., 2011; Distefano et al., 2009; Selkowitz et al., 2013) Though DKV has been targeted in functional exercises(Emamvirdi et al., 2019; Hopper et al., 2017; Lee et al., 2022; Myer et al., 2005), high loads would produce unsafe multi-joint kinematics, and these protocols target DKV rather through the mechanisms of motor learning. Our laboratory recently has also been investigating functional vs. non-functional abductor training, and we already have preliminary data (n = 20) showing that in the functional exercise the abductors can be loaded only up to 15-20% of MVIC, which participants can resist during a bilateral squat drill, which means very low intensity. Still, to your recommendation, we put this issue into the limitation section, using your sentences.

**Comment 3a (Statistical Analysis and Interpretation)**

Interaction Effects: The key finding of a significant group-by-time interaction for DKV during the SLCMJ (p < 0.05) is well-presented. However, the interpretation of the non-significant interaction for DKV during the landing task (p > 0.05) needs to be more nuanced. The paper states "eccentric vs. concentric training reduced DKV in single leg CMJ more than in single leg drop landing." This is accurate, but it should be emphasized that the training did not differentially affect the landing task. The discussion should explore potential reasons for this task-dependent adaptation more deeply.

**Response to comment 3a**

Thank you for calling our attention to this issue, which was really missing from the discussion. We now explore potential reasons in the discussion.

**Comment 3b**

Correlation Analysis: The lack of correlation between strength gains and DKV reduction is a critical finding that challenges the study's primary hypothesis. The current discussion offers plausible reasons (e.g., timing of activation vs. maximal strength, differential muscle usage). This section should be expanded, as it points toward neuromuscular control rather than raw strength being the primary driver of the observed improvements. The authors should consider whether the sample size (n=20) was sufficient to detect a moderate correlation, and note this as a limitation for the correlation analysis.
**Response to comment 3b**

In this section we intended to avoid suggestive over-explanations for the lack of correlation. The strongest reason is probably the low number of participants. A post hoc power analysis (G*Power) indicated that with a sample size of n = 20, the statistical power to detect a moderate correlation (r = 0.30, α = 0.05) was approximately 0.25, suggesting that the study may have been underpowered to detect such effects, and 82 participants would have been needed. We discussed about it extensively with our co-authors and decided to remove the correlation issue from the entire manuscript (including hypothesis, results, and specific section in the discussion).

**Comment 4 (Clarity of Methods - Work Equilibration)**

The description of equating mechanical work between groups needs clarification. The authors state that concentric subjects performed an additional 5th set to match the work of the eccentric subjects. This implies that the training stimulus was not identical in volume. It also suggests that the "maximal effort" in the concentric group's 5th set may have been performed under significant fatigue. Please provide more detail on the timing of this 5th set and how the total work was calculated and matched session-by-session.

**Response to comment 4**

Using number of sets and repetitions (indicators are volume) are though useful training variables in practice to quantify training load, alone, they do not precisely characterize the magnitude of mechanical stimulus in a given muscle. Mechanical work is a key stimulus in short-term strength gains (Hortobágyi & DeVita, 2000), and work-matched designs (considering both baseline strength levels and continuous monitoring of mechanical work) have been extensively used in scientific setups, while, scientists still control for the number of repetitions within a set (Hortobágyi & DeVita, 2000; Hyldahl et al., 2014; Váczi et al., 2014; Vieira et al., 2018). Regarding your comment on fatigue, we checked the training data, and we compared the mechanical work performed in sets 4 and 5 during the first and the last training sessions in the concentric group. Set 5 was +2.6% greater than set 4 in the first session, and set 5 was −1.06% smaller then set 4 in the last session, of which only the former reached statistical significance. This suggests no effect of fatigue in set 5 in the concentric group. For the clarification of the matching design, we edited the section.

Minor Concerns:

**Comment 5 (Terminology and Grammar)**

"Dynamic knee valgus": This term is correctly used, but the manuscript would benefit from a brief operational definition early in the methods (e.g., "defined as the frontal plane projection angle...").

"countermovement jump": Please ensure this term is consistently capitalized (CMJ) after its first use, as it appears in lowercase in several places (e.g., Abstract line 40, Results line 256).

Abstract Line 37: "dynamic knee valgus" should be capitalized as "Dynamic knee valgus" to start the sentence.

**Response to comment 5**

Now we provide the definition of DKV in the first sentence.

Thank you for noticing the errors regarding the CMJ. In the abstract, we avoided the use of abbreviations on purpose. CMJ is first capitalized in the introduction. Everywhere else, now we use consistently SLCMJ in the results section.

**Comment 6 (Data Presentation)**

Table 2: The table is very dense. Consider splitting the ANOVA results into a separate table or using symbols (e.g., *, †) directly in the table to denote significant main effects and interactions for easier readability. The column headers for the ANOVA results are also difficult to follow.

**Response to comment 6**

We now use symbols to denote significances. Regarding the ANOVA column headers, would you please clarify what is difficult to follow? We show ANOVA time main effects and interactions, regularly used as ANOVA test results.

**Comment 7 (Specific Discussion Points)**

Lines 307-310: The statement regarding the lack of effects in previous studies should be tempered. The current study uses a different population, training method (isokinetic), and outcome measure, making direct comparisons difficult.
**Response to comment 7**

Thank you, we have tempered the statement using your words.

**Comment 8 (Conclusion)**

The conclusion in the abstract and main text ("improves dynamic knee valgus and vertical jump performance") is too broad. It should specify that it improves DKV in a task-dependent manner (favoring SLCMJ) and improves jump performance similarly to concentric training.
**Response to comment 8**

Your statement is not exactly true because eccentric training improved DKV in both tests, and not in a “task-dependent manner”. Still, we modified the concluding sentences as follows: “High-intensity eccentric versus concentric hip abductor strength training was superior in dynamic knee valgus improvement measured during single-leg countermovement jump but not during drop landings in asymptomatic young women, while both training modalities improved single leg countermovement jump performance.”

Questions for the Authors:

**Comment 9**

What was the rationale for choosing a 4-week intervention? Was a power analysis conducted to determine the sample size of 20, and if so, what was the expected effect size for the primary outcome (DKV change)?

**Response to comment 9**

We selected the 4-week intervention period based on the study showing that „such a short-term intervention is known to produce changes in lower limb kinematics during sidestep cutting, a task closely associated with ACL injury risk, in female athletes” (Nijmeijer et al., 2024). Because of the uniqueness of the protocol (maximal effort ECC and CON contractions) and the inclusions, after n=20, we performed post-hoc power analysis (G*Power), eventually showing very high power values for the primary outcomes (DKV during single leg CMJ = 1.0, DKV during single leg landing = 0.99, eccentric hip abduction torque = 1.0, concentric hip abduction torque = 1.0.). Power values are now presented in the manuscript. (Note: we provided all open raw data in the repositorium along with the manuscript submission.)

**Comment 10**

Were the participants blinded to their group allocation (eccentric vs. concentric)? If not, how might this have influenced the results?

**Response to comment 10**
In the experimental procedure section we state that “Participants were unaware about the identity of their matched partner in the other experimental group”. They were even unaware about that they had matched partners. Additionally, no specific details about the biomechanical and adaptation mechanism of eccentric and concentric contractions were given.

**Comment 11**

Could the "task-dependent" improvement in the SLCMJ be partially explained by a learning effect specific to the jump task, given that the training did not involve a similar plyometric movement?
 **Response to comment 11**

Improvements in SLCMJ was our expectation, based on one study showing similar improvements (Giustino et al. 2022 (Giustino et al., 2022), referenced in discussion), and based on our own investigations showing that hip abductor strength (Sebesi et al., 2021) and/or its activation (Vadász et al., 2022) are associated with CMJ performance. Three repetitions of CMJs with 4-weeks of washout before the re-test, in our opinion, would have not revealed a learning effect, at least we have no information on such an effect.

**Responses to Reviewer #4 Comments**

Dear Reviewer, thank you for reviewing our manuscript.

**Comment 1**

I suggest softening the conclusions and avoiding statements that imply injury-prevention benefits. Considering the pilot design, the small sample size, the use of DKV as a surrogate outcome, and the lack of muscle activation measures, the results should be interpreted as suggestive findings showing a task-specific effect on DKV rather than evidence of reduced ACL injury risk.

**Response to comment 1**

You are absolutely right, but the term “ACL tear” is used only at the beginning of the discussion, and is only used for showing its mechanistic link to DKV. Nowhere is ACL injury risk mentioned in later paragraphs nor is it mentioned in the conclusions.

**References**

Boren, K., Conrey, C., Le Coguic, J., Paprocki, L., Voight, M., & Robinson, T. K. (2011). Electromyographic analysis of gluteus medius and gluteus maximus during rehabilitation exercises. *International Journal of Sports Physical Therapy*, *6*(3), 206–223.

Distefano, L. J., Blackburn, J. T., Marshall, S. W., & Padua, D. A. (2009). Gluteal Muscle Activation During Common Therapeutic Exercises. *Journal of Orthopaedic & Sports Physical Therapy*, *39*(7), 532–540. https://doi.org/10.2519/jospt.2009.2796

Emamvirdi, M., Letafatkar, A., & Khaleghi Tazji, M. (2019). The Effect of Valgus Control Instruction Exercises on Pain, Strength, and Functionality in Active Females With Patellofemoral Pain Syndrome. *Sports Health*, *11*(3), 223–237. https://doi.org/10.1177/1941738119837622

Giustino, V., Messina, G., Patti, A., Padua, E., Zangla, D., Drid, P., Battaglia, G., Palma, A., & Bianco, A. (2022). Effects of a Postural Exercise Program on Vertical Jump Height in Young Female Volleyball Players with Knee Valgus. *International Journal of Environmental Research and Public Health*, *19*(7), 3953. https://doi.org/10.3390/ijerph19073953

Hopper, A. J., Haff, E. E., Joyce, C., Lloyd, R. S., & Haff, G. G. (2017). Neuromuscular Training Improves Lower Extremity Biomechanics Associated with Knee Injury during Landing in 11–13 Year Old Female Netball Athletes: A Randomized Control Study. *Frontiers in Physiology*, *8*, 883. https://doi.org/10.3389/fphys.2017.00883

Hortobágyi, T., & DeVita, P. (2000). Favorable neuromuscular and cardiovascular responses to 7 days of exercise with an eccentric overload in elderly women. *The Journals of Gerontology. Series A, Biological Sciences and Medical Sciences*, *55*(8), B401-410. https://doi.org/10.1093/gerona/55.8.b401

Hyldahl, R. D., Olson, T., Welling, T., Groscost, L., & Parcell, A. C. (2014). Satellite cell activity is differentially affected by contraction mode in human muscle following a work-matched bout of exercise. *Frontiers in Physiology*, *5*. https://doi.org/10.3389/fphys.2014.00485

Lee, J. H., Shin, K. H., Han, S.-B., Sun Hwang, K., Lee, S. J., & Jang, K.-M. (2022). Prospective comparative study between knee alignment-oriented static and dynamic balance exercise in patellofemoral pain syndrome patients with dynamic knee valgus. *Medicine*, *101*(37), e30631. https://doi.org/10.1097/MD.0000000000030631

Li, J., & Qian, H. (2025). Biomechanical effects of different change of direction angles on lower limb joint load and injury risk in soccer players. *Scientific Reports*, *15*(1), 45123. https://doi.org/10.1038/s41598-025-33102-7

Myer, G. D., Ford, K. R., Palumbo, J. P., & Hewett, T. E. (2005). Neuromuscular Training Improves Performance and Lower-Extremity Biomechanics in Female Athletes. *The Journal of Strength and Conditioning Research*, *19*(1), 51. https://doi.org/10.1519/13643.1

Nijmeijer, E. M., Kempe, M., Elferink-Gemser, M. T., & Benjaminse, A. (2024). Observe, Practice, and Improve? Enhancing Sidestep Cutting Execution in Talented Female Soccer Players: A Four-Week Intervention Program With Video Instruction. *Journal of Strength & Conditioning Research*, *38*(8), e430–e439. https://doi.org/10.1519/JSC.0000000000004796

Sebesi, B., Fésüs, Á., Varga, M., Atlasz, T., Vadász, K., Mayer, P., Vass, L., Meszler, B., Balázs, B., & Váczi, M. (2021). The Indirect Role of Gluteus Medius Muscle in Knee Joint Stability during Unilateral Vertical Jump and Landing on Unstable Surface in Young Trained Males. *Applied Sciences*, *11*(16), 7421. https://doi.org/10.3390/app11167421

Selkowitz, D. M., Beneck, G. J., & Powers, C. M. (2013). Which Exercises Target the Gluteal Muscles While Minimizing Activation of the Tensor Fascia Lata? Electromyographic Assessment Using Fine-Wire Electrodes. *Journal of Orthopaedic & Sports Physical Therapy*, *43*(2), 54–64. https://doi.org/10.2519/jospt.2013.4116

Váczi, M., Nagy, S. A., Kőszegi, T., Ambrus, M., Bogner, P., Perlaki, G., Orsi, G., Tóth, K., & Hortobágyi, T. (2014). Mechanical, hormonal, and hypertrophic adaptations to 10weeks of eccentric and stretch-shortening cycle exercise training in old males. *Experimental Gerontology*, *58*, 69–77. https://doi.org/10.1016/j.exger.2014.07.013

Vadász, K., Varga, M., Sebesi, B., Hortobágyi, T., Murlasits, Z., Atlasz, T., Fésüs, Á., & Váczi, M. (2022). Frontal Plane Neurokinematic Mechanisms Stabilizing the Knee and the Pelvis during Unilateral Countermovement Jump in Young Trained Males. *International Journal of Environmental Research and Public Health*, *20*(1), 220. https://doi.org/10.3390/ijerph20010220

Vieira, A., Blazevich, A., Souza, N., Celes, R., Alex, S., Tufano, J. J., & Bottaro, M. (2018). Acute changes in muscle thickness and pennation angle in response to work-matched concentric and eccentric isokinetic exercise. *Applied Physiology, Nutrition, and Metabolism*, *43*(10), 1069–1074. https://doi.org/10.1139/apnm-2018-0055
